# Acute Demyelinating Encephalomyelitis Following Measles Infection Due to Vaccine Failure: A Case Report

**DOI:** 10.5811/cpcem.2021.2.49481

**Published:** 2021-03-24

**Authors:** Robert J. McMickle, Lauren Fryling, Ross J. Fleischman

**Affiliations:** Harbor-UCLA Medical Center, Department of Emergency Medicine, Torrance, California

**Keywords:** Measles, acute demyelinating encephalomyelitis, vaccine failure, case report

## Abstract

**Introduction:**

Local outbreaks of measles infection are primarily mediated by international travel of persons from endemic areas, with subsequent spread of the virus via undervaccinated populations. Recent resurgences of measles in communities where vaccination rates are non-ideal secondary to philosophical objections require the emergency physician to more routinely consider the diagnosis. In cases of measles complicated by acute encephalitis or encephalopathy, the diagnosis can be especially difficult to make due to lack of a reliable primary historian.

**Case report:**

Here we present a case of altered mental status and new-onset bilateral lower extremity weakness in a fully vaccinated young woman diagnosed with measles infection caused by acute disseminated encephalomyelitis in the setting of vaccine failure.

**Conclusion:**

Despite a documented history of immunization, acute measles infection and its uncommon sequelae are possible. Recognizing vaccine failure and appropriately isolating patients are of paramount importance.

## INTRODUCTION

Measles, or rubeola, is a highly contagious viral illness spread via larger respiratory droplets (ie, cough, sneeze) as well as smaller airborne transmission (ie, breathing, speaking).^1^ Patients typically develop symptoms after an incubation period of 10–12 days after initial exposure (range 7–21 days).^1^ Patients will first develop fever followed by the classic triad of cough, coryza, and conjunctivitis a few days before a rash appears.^1^ The prototypical rash starts on the face and spreads to the extremities, eventually forming a confluence. Patients are considered contagious for 1–2 days prior to symptom onset and for four days after the initial appearance of the rash.^1^,^2^ Rash is common, although immunocompromised patients including those with leukemias, lymphomas, or human immunodeficiency virus may never develop a rash.^1^

The current standard two-dose vaccination schedule was established in response to primary vaccine failure, and in total provides up to 97–99% protection against measles throughout life, although the incidence of disease is heavily impacted by the effectiveness of herd immunity. Primary vaccine failure is a relatively rare event, occurring in roughly 1% of patients due to failure of a patient to generate antibodies from antigenic stimuli.^4^

Complications from measles infection are common, occurring in upwards of 40% of all measles infection.^5^ Serious neurologic complications occur in roughly 1 in 1000 patients presenting with measles, including isolated measles encephalitis or meningitis, and acute demyelinating encephalomyelitis (ADEM), which typically presents as acute multifocal neurologic symptoms associated with encephalopathy, paraparesis or quadriparesis, sensory deficits, oculomotor deficits, and/or dysarthria.^6^ Severe cases can present as coma, seizure, or obtundation and carry a high rate of morbidity and mortality of 10–20%.^2^,^3^

## CASE REPORT

A 25-year-old otherwise healthy, fully-vaccinated young woman working as a nursing assistant in an urgent care clinic presented to the emergency department (ED) via ambulance for worsening mental status over the day preceding presentation. The patient had one week of a viral prodrome including rhinorrhea, congestion, cough, malaise, and facial rash. Her symptoms progressed until the morning of admission, when the patient’s partner noticed her to be more confused with repetitive speech, disorientation, and unresponsiveness. The partner also noticed decreased movement in her lower extremities and an inability to ambulate beginning earlier in the day of admission.

It was revealed that the patient had a known exposure to a measles-positive patient (an international traveler from an endemic region) whom she encountered in the clinic where she was employed. Within one week of exposure to the patient, she developed fever and rash beginning on her face, and spreading down her neck to her chest and arms. Serological titers were drawn one week prior to presentation by local department of public health officials and she was found to have low immuno-globulin G (IgG) titers. Booster vaccination was deferred at the time due to the patient having active fever.

On arrival to the ED, our patient was immediately placed in an airborne isolation room. She was tachycardic to 120 beats per minute with a low-grade fever to 38°C. She was alert but oriented only to her name, and was speaking nonsensically. On exam she was observed to have a maculopapular rash on her face extending to her neck and chest. She was unable to cooperate with a complete neurologic exam to assess for motor function, but withdrew only weakly to painful stimuli in the bilateral lower extremities. Deep tendon reflexes were normal. She was given a two-liter bolus of normal saline, and was empirically started on meningitic dosing of vancomycin and ceftriaxone. With no preceding oral lesions or history of herpes simplex infection, addition of acyclovir was deferred.

Initial white blood cell count was 13.4 K/cubic millimeters (mm^3^) (normal range 4.5–10.0 K/mm^3^), with 79% (44% – 71%) neutrophils; hemoglobin 13.6 grams per deciliter (g/dL) (12 – 14.6 g/dL); platelet count 256 K/ mm^3^ (160 – 360 K/ mm^3^); sodium 136 millimoles per liter (mmol/L) (136 – 144 mmol/L); potassium 4.3 mmol/L (3.6–5.1 mmol/L); bicarbonate 20 mmol/L (22 – 32 mmol/L); blood urea nitrogen 37 milligrams (mg/dL) (8–20 mg/dL); creatinine 2.0 mg/dL (0.44–1.03 mg/dL); glucose 148 mg/dL (41–118 mg/dL); aspartate transaminase 65 units per liter (U/L) (15–41 U/L); alanine transaminase 47 U/L (7–35 U/L); and bilirubin 1.0 mg/dL (0.3 – 1.2 mg/dL).

A lumbar puncture was performed in the ED, with clear cerebrospinal fluid (CSF) and an opening pressure of 16 centimeters (cm) water (H_2_O) (normal range 7 – 18 cm H_2_O); nucleated cell count 134/ mm^3^ (0 – 5/ mm^3^), with 67% segmented neutrophils (0 – 6%); 23% lymphocytes (40 – 80%); 8% monocytes/histiocytes (no reference range available); and 2% plasma cells (no reference range available). Her CSF glucose was 71 mg/dL (40–75 mg/dL), and CSF protein was 70mg/dL (15–45 mg/dL). A CSF Gram stain and CSF viral polymerase chain reaction (PCR) panel (not including measles virus) were negative.

**CPC-EM Capsule**

What do we already know about this clinical entity?

*Measles vaccine failure is an uncommon yet possible occurrence, and acute measles infection in older individuals may present as an undifferentiated encephalitis*.

What makes this presentation of disease reportable?

*Rare and severe manifestations of measles including neurologic complications such as acute demyelinating encephalomyelitis are possible despite vaccination*.

What is the major learning point?

*Emergency providers cannot exclude measles in vaccinated individuals presenting with a prototypical viral exanthem or neurologic sequelae of disease*.

How might this improve emergency medicine practice?

*Early isolation and consideration of the diagnosis of measles can curb spread in communities experiencing outbreaks due to philosophical objections to vaccination*.

The patient was admitted to the internal medicine service with infectious disease consultation. Measles IgM and IgG were both elevated in sputum and plasma. She was treated with intramuscular vitamin A 200,000 international units for two days and continued on meningitis dosing of antibiotics until CSF cultures proved negative. Her mental status improved over the subsequent two days with a return to baseline, but she continued to have bilateral lower extremity weakness and new acute urinary retention. She was diagnosed with ADEM after magnetic resonance imaging (MRI) and nerve conduction studies were consistent with the diagnosis. She was started on high-dose methylprednisolone (30mg/kilogram) for five days with gradual improvement in symptoms. The patient was discharged on hospital day five with significantly improved neurologic function, although still not independently ambulatory and requiring an indwelling urinary catheter.

## DISCUSSION

Since its initial eradication in the United States in 2000, measles has had multiple resurgent outbreaks occurring yearly since 2010.^7^ A record-high 1282 cases confirmed in 31 states were observed throughout 2019, primarily via travel-related introductions of virus and spread through undervaccinated subpopulations in urban areas or healthcare settings.^7^,^8^ In light of this resurgence, even persons with documented vaccination history may be at risk of acquiring infection and may present with severe features.

A febrile exanthem and a CSF pleocytosis in encephalopathic patients can suggest a viral cause, and suspicion should be raised in patients with known exposures or who live or work in high-risk environments (ie, healthcare). Emergency providers will often presume a diagnosis of measles based on clinical criteria in the absence of viral studies. Measles-specific IgM antibody and measles ribonucleic acid by real-time PCR in respiratory specimens and a serum sample and throat swab should be obtained at first contact if suspicion is high.^9^

The mainstay of treatment for measles infection remains supportive. In this case vitamin A, which is known to decrease morbidity and mortality in children with measles by replenishing stores of vitamin A lost via destruction of epithelial surfaces by the virus,^10^ was administered, and the patient’s mental status recovery was sufficient enough to not warrant additional therapy with ribavirin or intravenous immunoglobulin. Administration of the measles-mumps-rubella vaccine within 72 hours of exposure or immunoglobulin within six days of exposure can be given in the ED as well. Patients should be placed as soon as possible in a single-patient airborne isolation room to prevent spread to the healthcare team or other patients. They should remain isolated for four days after they develop a rash.

Acute demyelinating encephalomyelitis is considered a diagnosis of exclusion. Magnetic resonance imaging of spinal series and brain with gadolinium should be obtained if suspicion for ADEM is high and may demonstrate patchy hyperintense lesions and edema throughout the brain and spinal cord. In this case, an MRI demonstrated leptomeningeal enhancement in the thoracic spine. High-dose solumedrol (30mg/kg) was administered for five days with subsequent improvement of patient’s bilateral lower extremity weakness. Intravenous immunoglobulin and plasma exchange may also be employed in non-improving patients,^6^ although typically these are not initiated in the ED without infectious disease consultation.

## CONCLUSION

Even in patients with a documented history of immunization, acute measles infection and its uncommon sequelae are possible. The emergency provider may be tempted to use history of vaccination as a screening tool to exclude such illnesses in their differential diagnosis; however, it is important for initial providers to be aware that vaccine failure, although uncommon, is possible and should be considered in the multiple vaccine-preventable illnesses including varicella, influenza, and *Haemophilus influenzae*. In addition to making an accurate diagnosis to initiate appropriate therapy, the initial evaluation by emergency providers must also include appropriately isolating suspect patients to prevent spread to the healthcare team or other patients.
